# Supplementary material for: Technological Frontiers in Brain Cancer: A Systematic Review and Meta-Analysis of Hyperspectral Imaging in Computer-Aided Diagnosis Systems
Source: Diagnostics (Basel). 2024 Aug 28;14(17):1888. doi: 10.3390/diagnostics14171888 (PMC11394276; doi:10.3390/diagnostics14171888)
Supplement: Supplementary file 1 [file diagnostics-14-01888-s001.zip › diagnostics-3113737-supplementary.pdf]

diagnostics

MDPI

Review

Technological Frontiers in Brain Cancer: A Meta-Analysis of Hyperspectral Imaging in Computer-Aided Diagnosis Systems: Supplementary Material

Joseph-Hang Leung [1], Riya Karmakar [2], Arvind Mukundan [2], Wen-Shou Lin [3,*], Fathima Anwar [5], and Hsiang-Chen Wang [2,4,6,*]

[1] Department of Radiology, Ditmanson Medical Foundation Chia-yi Christian Hospital, Chia-yi City, Taiwan; 01289@cych.org.tw (J.-H.L)

[2] Department of Mechanical Engineering, National Chung Cheng University, 168, University Rd., Min Hsiung, Chia Yi 62102, Taiwan; d09420003@ccu.edu.tw (A.M.); karmakarriya345@gmail.com (R.K.)

[3] Neurology Division, Department of Internal Medicine, Kaohsiung Armed Forces General Hospital, 2, Zhongzheng 1st.Rd., Lingya District, Kaohsiung City 80284, Taiwan; linvincent1009@gmail.com (W.-S.L.)

[4] Department of Medical Research, Dalin Tzu Chi Hospital, Buddhist Tzu Chi Medical Foundation, No. 2, Minsheng Road, Dalin, Chiayi, 62247 Taiwan

[5] Faculty of Allied Health Sciences, The University of Lahore, 1-Km Defense Road, Lahore, Punjab, Pakistan; fatimanwarx@gmail.com (F.A)

[6] Director of Technology Development, Hitspectra Intelligent Technology Co., Ltd., 8F.11-1, No. 25, Chenggong 2nd Rd., Qianzhen Dist., Kaohsiung City 80661, Taiwan

[*] Correspondence: linvincent1009@gmail.com (W.-S.L.) and hcwang@ccu.edu.tw (H.-C.W.)

Abstract: Brain cancer is a substantial factor in the mortality associated with cancer, presenting difficulties in the timely identification of the disease. The precision of diagnoses is significantly dependent on the proficiency of radiologists and neurologists. Although there is potential for early detection with computer-aided diagnosis (CAD) algorithms, the majority of current research are hindered by their modest sample sizes. This meta-analysis aims to comprehensively assess the diagnostic test accuracy (DTA) of computer-aided design (CAD) models specifically designed for the detection of brain cancer utilizing hyperspectral (HSI) technology. We employ Quadas-2 criteria to choose seven papers and classify the proposed methodologies according on artificial intelligence method, cancer type, and publication year. In order to evaluate heterogeneity and diagnostic performance, we utilize Deeks' funnel plot, forest plot, and accuracy charts. The results of our research suggest that there is no notable variation among the investigations. The CAD techniques that have been examined exhibit a notable level of precision in the automated detection of brain cancer. However, the absence of external validation hinders their potential implementation in real-time clinical settings. This highlights the necessity for additional study in order toauthenticate CAD models for wider clinical applicability

Keywords: Brain Cancer; Hyperspectral imaging; Computer-Aided Diagnosis; Deek's funnel plot, Forest Plot, Meta-Analysis, Diagnostic test accuracy.

S1. Literature Search

Two authors independently searched the articles specifically on the web, google scholar's search engine to utilize in this study. Literature from the year of 2018 to 2024 was selected for this review. Duplications of any article was not considered. It was necessary to review the titles and abstracts of the identified articles to avoid irrelevancy of publications to the goal of this review. Moreover, to met the criteria of inclusion full-text reviews were done.

S1.1 Inclusion Criteria

This review intends to focus on studies that satisfies the established inclusion criteria:

1. Studies with conclusive numerical results such as dataset, sensitivity, accuracy, precision, and area under the curve (AUC).
2. Focused on hyperspectral imaging dealing with brain cancer detection.
3. published with in the last 6 years.
4. publication journal must be in the first quartile (Q1) and have an H-index greater than 50.
5. studies with prospective or retrospective design.
6. Studies must be written in English.

S1.2 Exclusion Criteria

1. This review will exclude studies that will fall under the following exclusion criteria:
2. studies with small-scale data.
3. studies including narrative, systematic review, and meta-analyses.
4. comments, proceedings, or study protocols.
5. conference papers.

S.1.3 Data Extraction, Primary Outcomes, and Additional Analyses

Two authors (F.A and A.M) performed the extraction and cross-checking of the data. The primary source of communication for data validation and inquires was by email. The majority of data collection in the meta-analysis was mostly about accuracy, sensitivity, and specificity of the diagnostic performance based on brain tumor imaging in each study. A systematic review process and diagnostic test accuracy (DTA) was utilized for synthesizing of each study. Additionally, a subgroup analysis made up of data's geographical origin was recorded. The type of vivo, type of CAD methods, and type of brain cancer was as well provided in the subgroup table for further analysis.

S.1.4 Study Inclusion

Upon searching in Google Scholar total of 10,600 results were distinguished. After considering the publishing years 4,060 articles were excluded, since this review will mainly focus on articles published in years of 2018 to 2024. Articles with full-text access were also considered, leaving up to 3710 articles to be excluded. A total of 2830 was left to be reviewed. Among these remaining articles was with incomplete data, narrative reviews and meta-analysis or comments and conference papers that are part of exclusion criteria. Consequently, 7 studies were included in this review. Supplementary figure 1 shows the flowchart of the selection process.

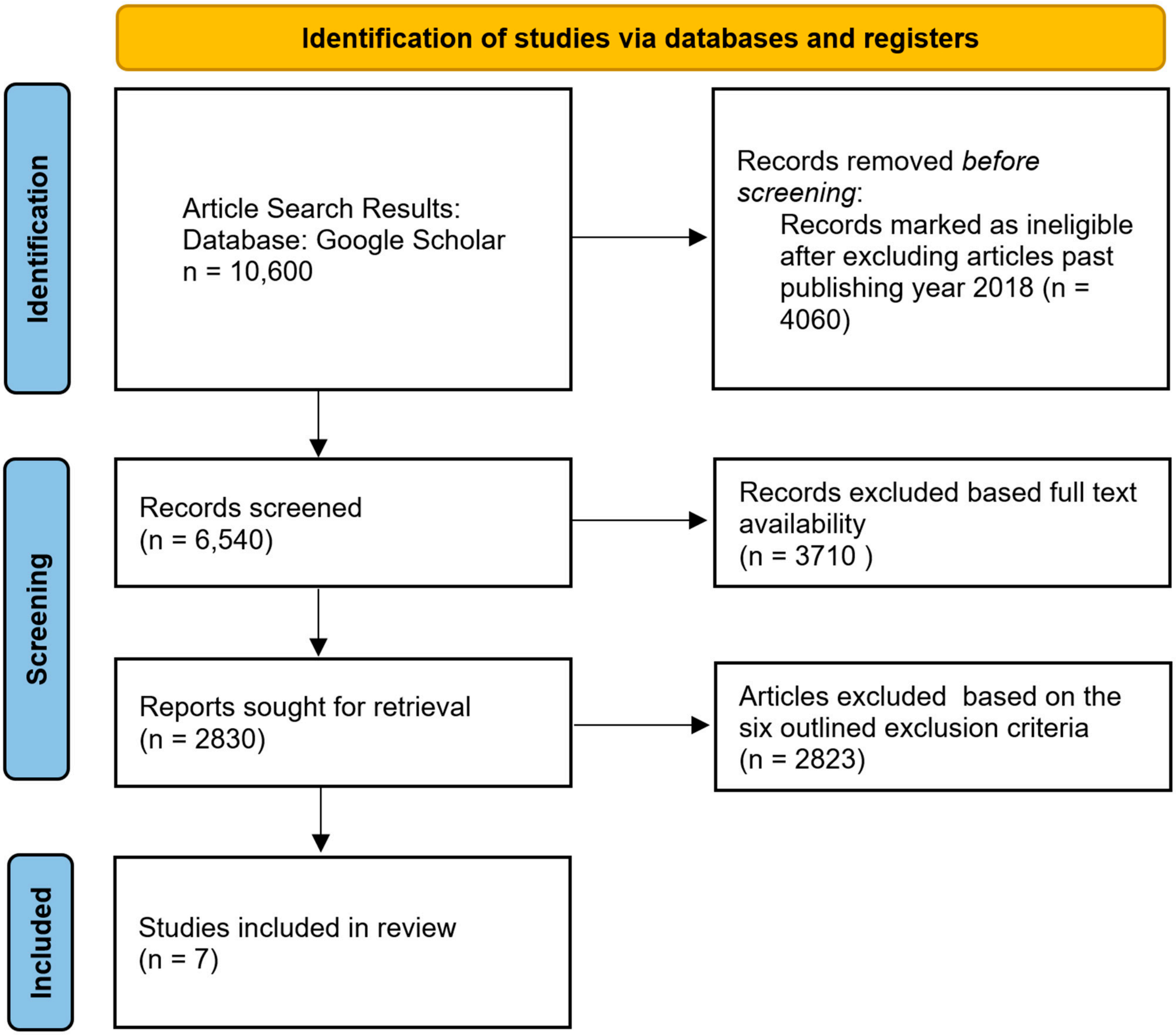

Figure S1. PRISMA 2020 flow diagram

S2. Quality Analysis

A precise and detailed information from a study to be reviewed is essential and considered to be a good quality for a precise inference. This precise conclusion improves CAD methods in data training and learning. In this study, a couple of esophageal cancer lesion images and involved esophageal cancer patients were the main contributor of data needed for training. Nonetheless, risk of bias and concerns regarding applicability must be used since not all studies in this review provided detailed description of the patient enrollment standard, index test, and reference standard. All studies were in "low risk" in terms of concerns regarding applicability. Maktabi et al. and Grigoroiu et al. were labelled as "unclear risk" in terms of index test due to the absence of primary outcomes as well as values for specificity and sensitivity. Study by Grigoroiu et al. also received "unclear risk" in reference standard and flow and timing due to the lack of supporting data for their stated average consistency in diagnostic accuracy.

S2.1 QUADAS-2

In this section QUADAS-2 results of seven studies are summarized for the review. It consists of applicability concerns and the level of risk of bias of the studies based on flow and timing, patient selection, reference standard, and index test. Each study was thoroughly reviewed under flow and timing, patient selection, reference standard, and index test for the risk of bias as well as under patient selection, reference standard, and index test for applicability concerns.

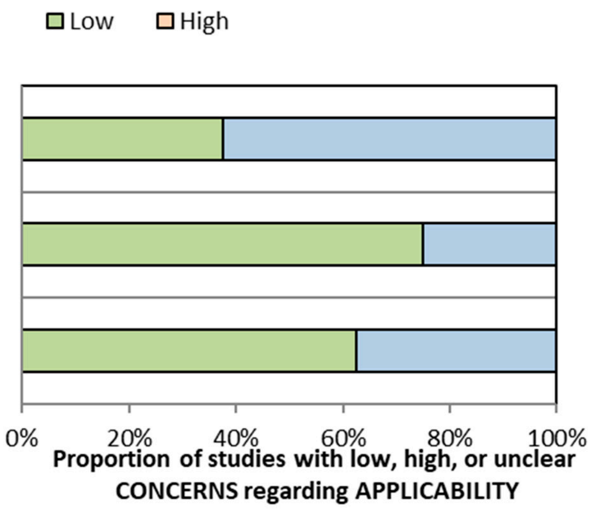

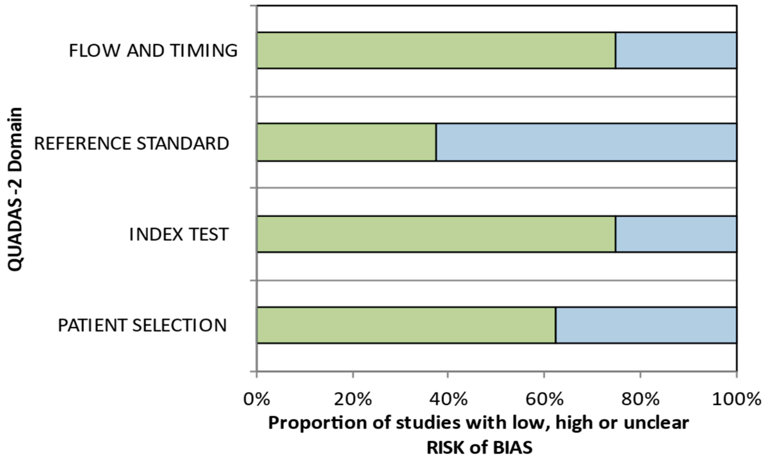

Figure S2.QUADAS-2 Domain

S3. Forest Plot

This section contains forest plots of sensitivity and specificity for different classifications involved in the study. The forest plot used the confidence level of 95% with upper and lower limit to explain the quality of the data involved in the study. The quality of the data can be interpreted in accordance with the line of no effect from forest plot. The line of no effect was calculated by deriving the average of the specificity and sensitivity of the data involved in each classification in this study. The overlapping of data on line indicated the data as a low performance data. By contrast, data away from line of no effect shows the high performance of the data.

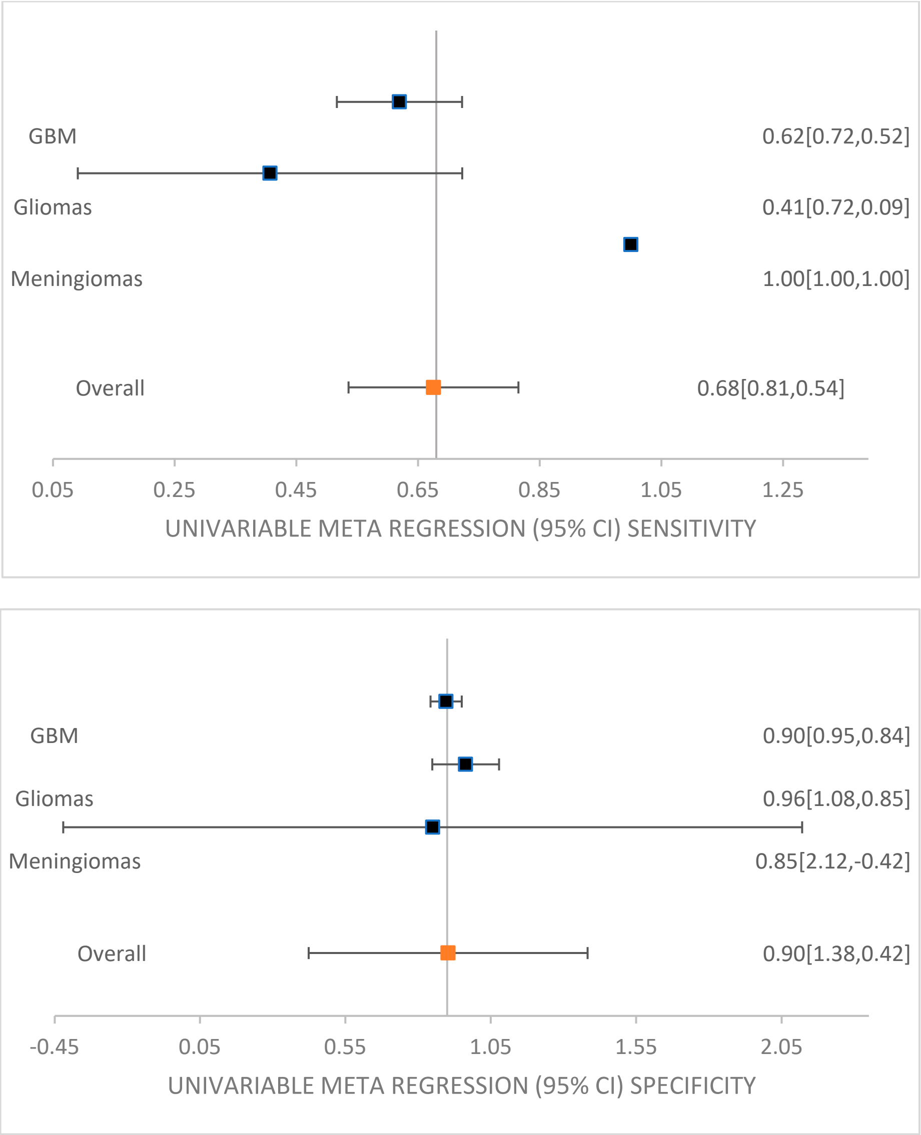

Figure S3. Sensitivity and Specificity Forest Plot (cancer types)

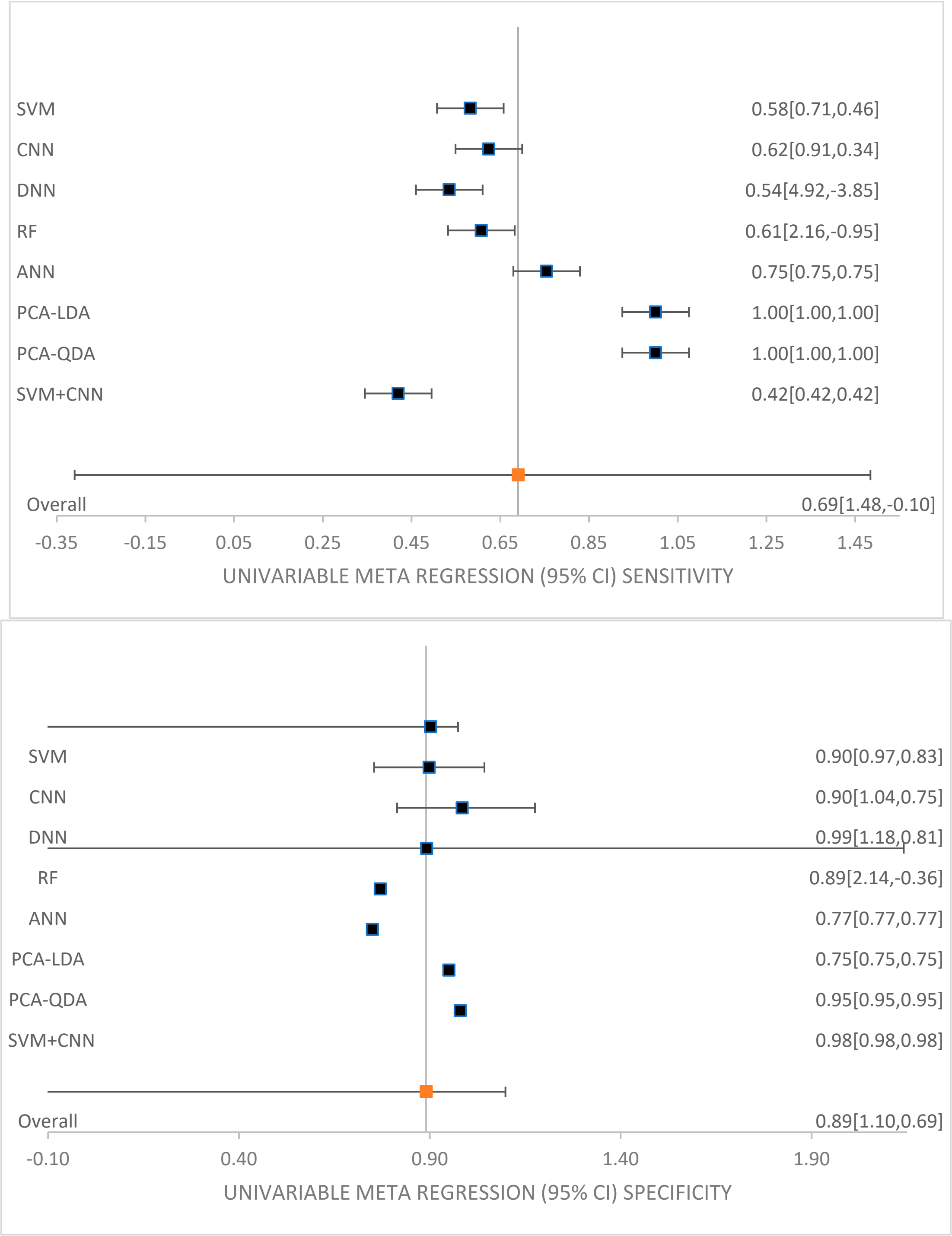

Figure S4. Sensitivity and Specificity Forest Plot (AI types)

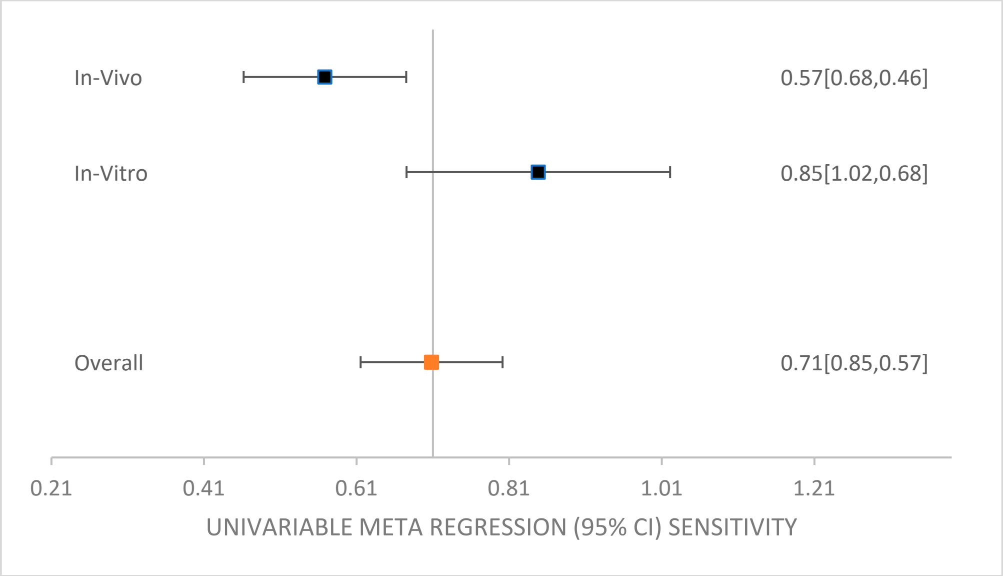

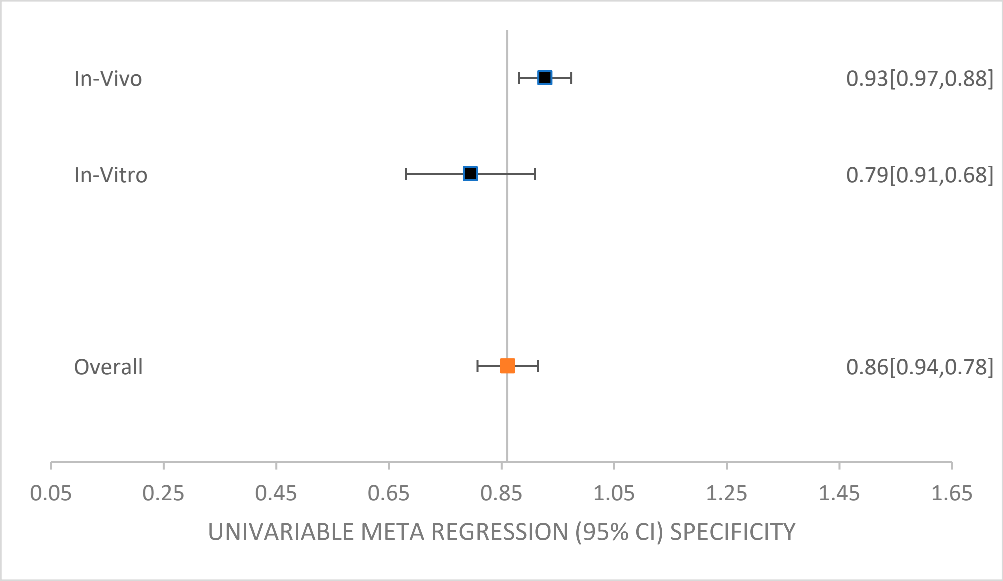

Figure S5.Sensitivity and Specificity Forest Plot (Vivo types)

S4. Deeks' Funnel Plot

This section contains the Deek's funnel plot for different classifications such as vivo, AI methods, published year and Cancer type. Purpose of this funnel plot is evaluation of publication bias. It comprises of square root of the dataset and the ratio of diagnostic odds. The regression line is also there to make sure the consideration of the meta-analytical estimate of the publication biases.

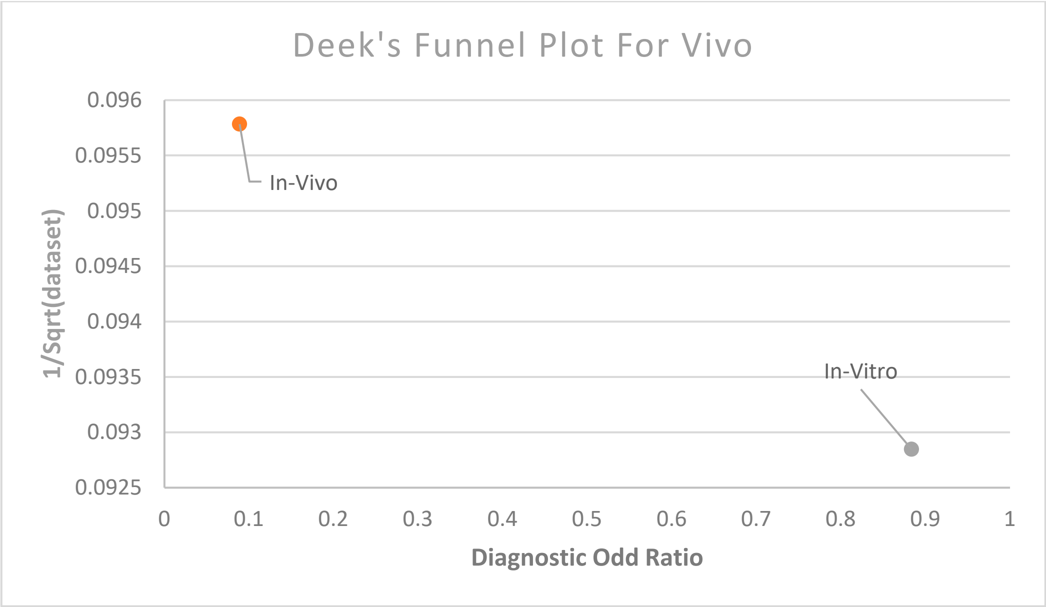

Figure S6. Deeks' Funnel Plot for Vivo

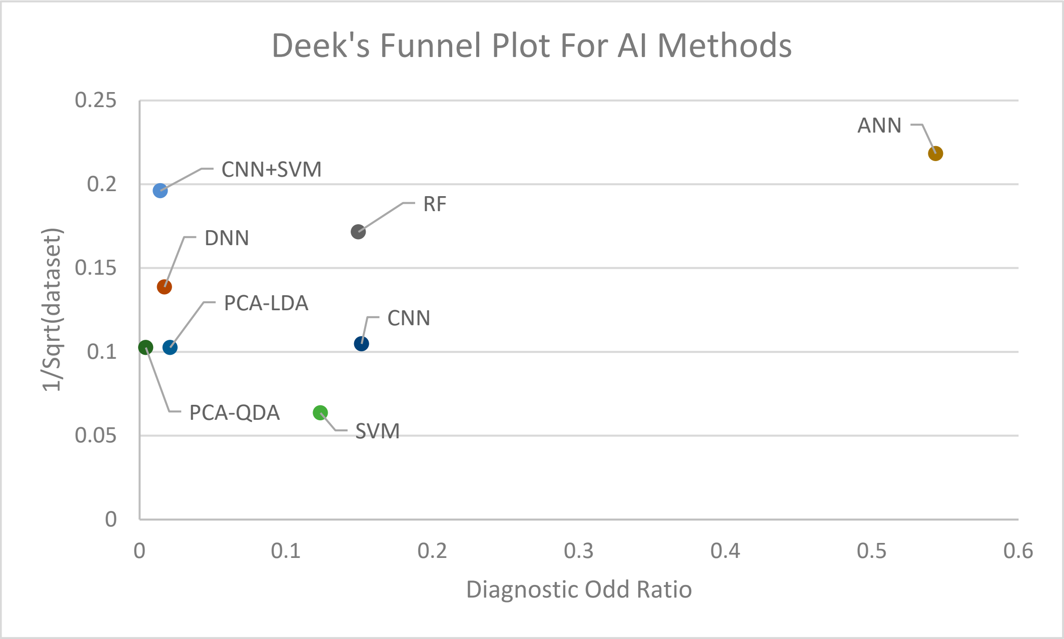

Figure S7. Deeks' Funnel Plot for AI Methods

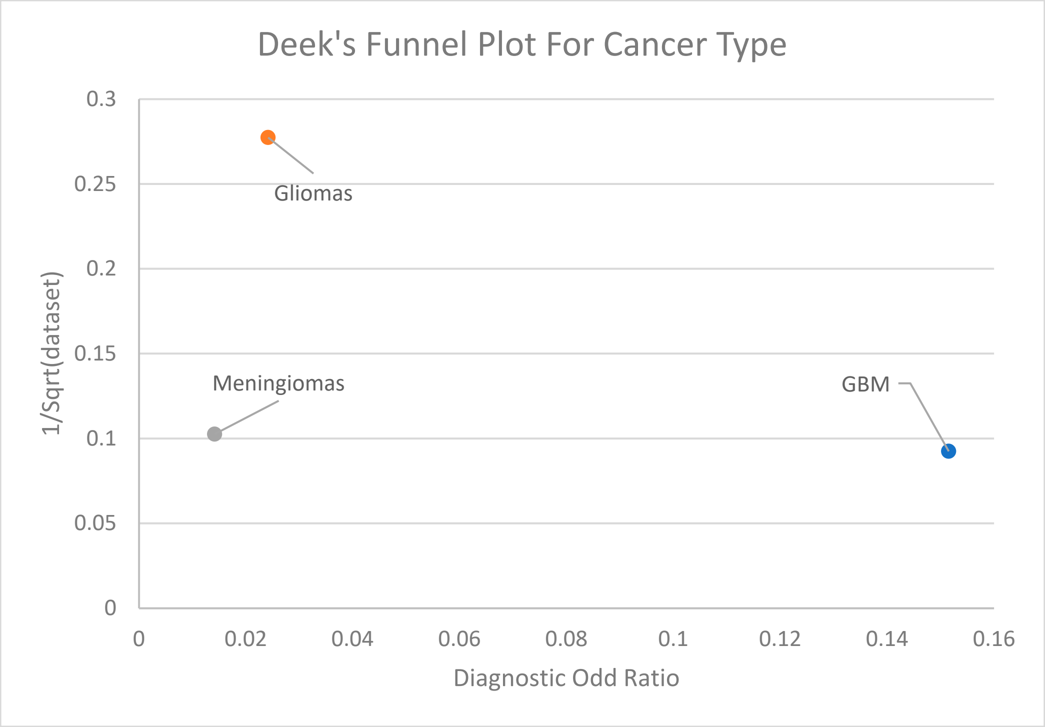

Figure S8. Deeks' Funnel Plot for Cancer type

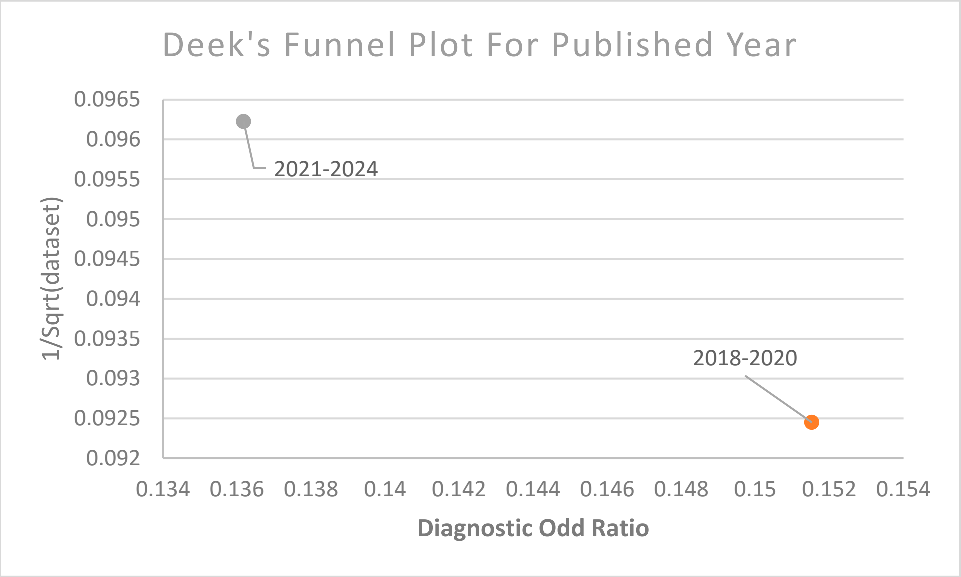

Figure S9. Deeks' Funnel Plot for published year

S5. Accuracy Chart

This section shows the accuracy chart of the data based on the different studies used in the review. It provides the better visualization and comparison of the data.

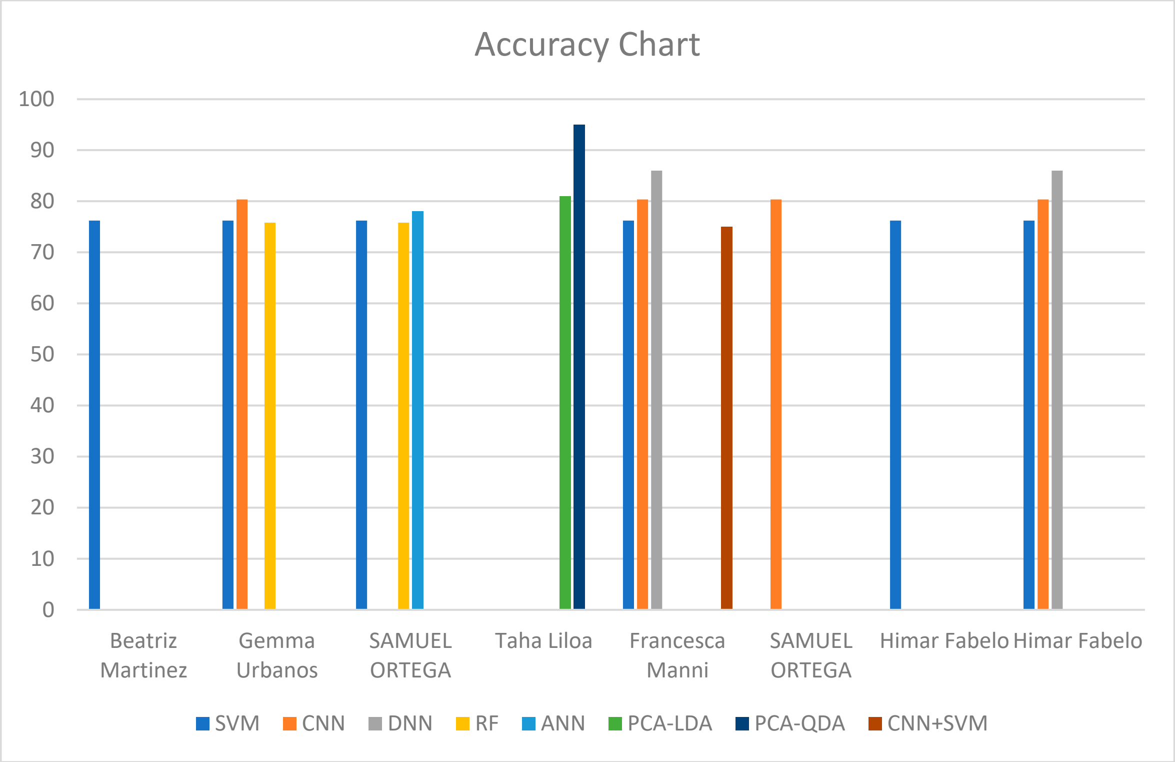

Figure S10. Accuracy Chart of Studies

S6. Summary of Computations for Forest Plots

The computations obtained from each plot are present in this section. It contains the mean and confidence level that is essential in plotting the forest plot.

Sensitivity of in-vivo	
Mean	56.846
Standard Error	5.09058472
Median	55.5
Mode	68
Standard Deviation	22.765787
Sample Variance	518.281057
Kurtosis	-0.26729
Skewness	-0.03709049
Range	85.62
Minimum	14
Maximum	99.62
Sum	1136.92
Count	20
Confidence Level(95.0%)	10.6547163
Upper CI	67.5007163
Lower CI	46.1912837

Specificity of in-vivo	
Mean	92.7005
Standard Error	2.222518779
Median	97.5
Mode	100
Standard Deviation	9.939406143
Sample Variance	98.79179447
Kurtosis	1.686770421
Skewness	-1.62907296
Range	32
Minimum	68
Maximum	100
Sum	1854.01
Count	20
Confidence Level(95.0%)	4.651785266
Upper CI	97.3522853
Lower CI	88.0487147

Sensitivity of in-vitro	
Mean	84.814
Standard Error	6.218280791
Median	75.69
Mode	100
Standard Deviation	13.90449855
Sample Variance	193.33508
Kurtosis	-3.28817831
Skewness	0.580746541
Range	27.06
Minimum	72.94
Maximum	100
Sum	424.07
Count	5
Confidence Level(95.0%)	17.26471527
Upper CI	102.078715
Lower CI	67.5492848

Specificity of in-vitro	
Mean	79.466
Standard Error	4.119374467
Median	77.03
Mode	#N/A
Standard Deviation	9.211201333
Sample Variance	84.84623
Kurtosis	3.090513468
Skewness	1.616285101
Range	24.03
Minimum	70.97
Maximum	95
Sum	397.33
Count	5
Confidence Level(95.0%)	11.43721708
Upper CI	90.903217
Lower CI	68.028783

Sensitivity of publishing year 2018-2020	
Mean	61.9495
Standard Error	4.91447826
Median	63
Mode	68
Standard Deviation	21.9782149
Sample Variance	483.041931
Kurtosis	0.24053447
Skewness	-0.56529047
Range	85.62
Minimum	14
Maximum	99.62
Sum	1238.99
Count	20
Confidence Level(95.0%)	10.2861212
Upper CI	72.2356212
Lower CI	51.6633788

Specificity of publishing year 2018-2020	
Mean	89.617
Standard Error	2.565548582
Median	95.8
Mode	100
Standard Deviation	11.47348206
Sample Variance	131.6407905
Kurtosis	-0.95777633
Skewness	-0.7985772
Range	32
Minimum	68
Maximum	100
Sum	1792.34
Count	20
Confidence Level(95.0%)	5.369754895
Upper CI	94.9867549
Lower CI	84.2472451

Sensitivity of publishing year 2021-2024	
Mean	64.4

Specificity of publishing year 2021-2024	
Mean	91.8

Standard Error	15.07929043
Median	48.5
Mode	100
Standard Deviation	33.71831846
Sample Variance	1136.925
Kurtosis	-2.65010201
Skewness	0.269187332
Range	74
Minimum	26
Maximum	100
Sum	322
Count	5
Confidence Level(95.0%)	41.86682212
Upper CI	106.266822
Lower CI	22.5331779

Standard Error	4.45421149
Median	95
Mode	99
Standard Deviation	9.959919678
Sample Variance	99.2
Kurtosis	2.814776275
Skewness	-1.67121441
Range	24
Minimum	75
Maximum	99
Sum	459
Count	5
Confidence Level(95.0%)	12.36687369
Upper CI	104.166874
Lower CI	79.4331264

Sensitivity of GBM	
Mean	61.9495
Standard Error	4.91447826
Median	63
Mode	68
Standard Deviation	21.9782149
Sample Variance	483.041931
Kurtosis	0.24053447
Skewness	-0.56529047
Range	85.62
Minimum	14
Maximum	99.62
Sum	1238.99
Count	20
Confidence Level(95.0%)	10.2861212
Upper CI	72.2356212
Lower CI	51.6633788

Specificity of GBM	
Mean	89.617
Standard Error	2.565548582
Median	95.8
Mode	100
Standard Deviation	11.47348206
Sample Variance	131.6407905
Kurtosis	-0.95777633
Skewness	-0.7985772
Range	32
Minimum	68
Maximum	100
Sum	1792.34
Count	20
Confidence Level(95.0%)	5.369754895
Upper CI	94.9867549
Lower CI	84.2472451

Sensitivity of gliomas	
Mean	40.66666667
Standard Error	7.339012952
Median	47.5
Mode	#N/A

Specificity of gliomas	
Mean	96.33333333
Standard Error	2.666666667
Median	99
Mode	99

Standard Deviation	12.71154331
Sample Variance	161.5833333
Kurtosis	#DIV/0!
Skewness	-1.71999944
Range	22.5
Minimum	26
Maximum	48.5
Sum	122
Count	3
Confidence Level(95.0%)	31.57722411
Upper CI	72.2438907
Lower CI	9.0894425

Standard Deviation	4.618802154
Sample Variance	21.33333333
Kurtosis	#DIV/0!
Skewness	-1.732050808
Range	8
Minimum	91
Maximum	99
Sum	289
Count	3
Confidence Level(95.0%)	11.47374061
Upper CI	107.807074
Lower CI	84.8595927

Sensitivity of Meningiomas	
Mean	100
Standard Error	0
Median	100
Mode	100
Standard Deviation	0
Sample Variance	0
Kurtosis	#DIV/0!
Skewness	#DIV/0!
Range	0
Minimum	100
Maximum	100
Sum	200
Count	2
Confidence Level(95.0%)	0
Upper CI	100
Lower CI	100

Specificity of Meningiomas	
Mean	85
Standard Error	10
Median	85
Mode	#N/A
Standard Deviation	14.14213562
Sample Variance	200
Kurtosis	#DIV/0!
Skewness	#DIV/0!
Range	20
Minimum	75
Maximum	95
Sum	170
Count	2
Confidence Level(95.0%)	127.0620474
Upper CI	212.062047
Lower CI	-42.062047

Sensitivity of SVM	
Mean	58.2372727
Standard Error	5.65668648
Median	57
Mode	#N/A
Standard Deviation	18.7611066
Sample Variance	351.979122

Specificity of SVM	
Mean	90.18
Standard Error	3.224455698
Median	91.2
Mode	100
Standard Deviation	10.6943097
Sample Variance	114.36826

Kurtosis	2.01466768	Kurtosis	0.3760211
Skewness	0.7225682	Skewness	-1.1577045
Range	73.62	Range	29.7
Minimum	26	Minimum	70.3
Maximum	99.62	Maximum	100
Sum	640.61	Sum	991.98
Count	11	Count	11
Confidence Level(95.0%)	12.6038829	Confidence Level(95.0%)	7.184535016
Upper CI	70.8411556	Upper CI	97.364535
Lower CI	45.6333898	Lower CI	82.995465

Sensitivity of CNN		*Specificity of CNN*	
Mean	62.41666667	Mean	89.83333333
Standard Error	11.22825058	Standard Error	5.617927653
Median	72	Median	97.5
Mode	#N/A	Mode	#N/A
Standard Deviation	27.50348463	Standard Deviation	13.76105616
Sample Variance	756.4416667	Sample Variance	189.3666667
Kurtosis	1.245786846	Kurtosis	-0.800029163
Skewness	-1.31182082	Skewness	-1.126782759
Range	74	Range	32
Minimum	14	Minimum	68
Maximum	88	Maximum	100
Sum	374.5	Sum	539
Count	6	Count	6
Confidence Level(95.0%)	28.86313699	Confidence Level(95.0%)	14.44134278
Upper CI	91.2798035	Upper CI	104.274676
Lower CI	33.5535297	Lower CI	75.3919906

Sensitivity of DNN		*Specificity of DNN*	
Mean	53.5	Mean	98.5
Standard Error	34.5	Standard Error	1.5
Median	53.5	Median	98.5
Mode	#N/A	Mode	#N/A
Standard Deviation	48.7903679	Standard Deviation	2.121320344
Sample Variance	2380.5	Sample Variance	4.5
Kurtosis	#DIV/0!	Kurtosis	#DIV/0!
Skewness	#DIV/0!	Skewness	#DIV/0!

Range	69
Minimum	19
Maximum	88
Sum	107
Count	2
Confidence Level(95.0%)	438.364063
Upper CI	491.864063
Lower CI	-384.864063

Range	3
Minimum	97
Maximum	100
Sum	197
Count	2
Confidence Level(95.0%)	19.0593071
Upper CI	117.559307
Lower CI	81.4406929

Sensitivity of RF	
Mean	60.72
Standard Error	12.22
Median	60.72
Mode	#N/A
Standard Deviation	17.28168973
Sample Variance	298.6568
Kurtosis	#DIV/0!
Skewness	#DIV/0!
Range	24.44
Minimum	48.5
Maximum	72.94
Sum	121.44
Count	2
Confidence Level(95.0%)	155.2698219
Upper CI	215.989821
Lower CI	-94.549821

Specificity of RF	
Mean	89.165
Standard Error	9.835
Median	89.165
Mode	#N/A
Standard Deviation	13.90879039
Sample Variance	193.45445
Kurtosis	#DIV/0!
Skewness	#DIV/0!
Range	19.67
Minimum	79.33
Maximum	99
Sum	178.33
Count	2
Confidence Level(95.0%)	124.9655236
Upper CI	214.130523
Lower CI	-35.800523

Sensitivity of ANN	
Mean	75.44
Standard Error	0
Median	75.44
Mode	#N/A
Standard Deviation	#DIV/0!
Sample Variance	#DIV/0!
Kurtosis	#DIV/0!
Skewness	#DIV/0!
Range	0
Minimum	75.44

Specificity of ANN	
Mean	77.03
Standard Error	0
Median	77.03
Mode	#N/A
Standard Deviation	#DIV/0!
Sample Variance	#DIV/0!
Kurtosis	#DIV/0!
Skewness	#DIV/0!
Range	0
Minimum	77.03

Maximum	75.44	Maximum	77.03
Sum	75.44	Sum	77.03
Count	1	Count	1
Confidence Level(95.0%)	#NUM!	Confidence Level(95.0%)	#NUM!
Upper CI	75.44	Upper CI	77.03
Lower CI	75.44	Lower CI	77.03

Sensitivity of PCA–LDA		*Specificity of PCA–LDA*	
Mean	100	Mean	75
Standard Error	0	Standard Error	0
Median	100	Median	75
Mode	#N/A	Mode	#N/A
Standard Deviation	#DIV/0!	Standard Deviation	#DIV/0!
Sample Variance	#DIV/0!	Sample Variance	#DIV/0!
Kurtosis	#DIV/0!	Kurtosis	#DIV/0!
Skewness	#DIV/0!	Skewness	#DIV/0!
Range	0	Range	0
Minimum	100	Minimum	75
Maximum	100	Maximum	75
Sum	100	Sum	75
Count	1	Count	1
Confidence Level(95.0%)	#NUM!	Confidence Level(95.0%)	#NUM!
Upper CI	100	Upper CI	75
Lower CI	100	Lower CI	75

Sensitivity of PCA–QDA		*Specificity of PCA–QDA*	
Mean	100	Mean	95
Standard Error	0	Standard Error	0
Median	100	Median	95
Mode	#N/A	Mode	#N/A
Standard Deviation	#DIV/0!	Standard Deviation	#DIV/0!
Sample Variance	#DIV/0!	Sample Variance	#DIV/0!
Kurtosis	#DIV/0!	Kurtosis	#DIV/0!
Skewness	#DIV/0!	Skewness	#DIV/0!
Range	0	Range	0
Minimum	100	Minimum	95
Maximum	100	Maximum	95
Sum	100	Sum	95

Count	1	Count	1
Confidence Level(95.0%)	#NUM!	Confidence Level(95.0%)	#NUM!
Upper CI	100	Upper CI	95
Lower CI	100	Lower CI	95

Sensitivity of CNN+SVM		*Specificity of CNN+SVM*	
Mean	42	Mean	98
Standard Error	0	Standard Error	0
Median	42	Median	98
Mode	#N/A	Mode	#N/A
Standard Deviation	#DIV/0!	Standard Deviation	#DIV/0!
Sample Variance	#DIV/0!	Sample Variance	#DIV/0!
Kurtosis	#DIV/0!	Kurtosis	#DIV/0!
Skewness	#DIV/0!	Skewness	#DIV/0!
Range	0	Range	0
Minimum	42	Minimum	98
Maximum	42	Maximum	98
Sum	42	Sum	98
Count	1	Count	1
Confidence Level(95.0%)	#NUM!	Confidence Level(95.0%)	#NUM!
Upper CI	42	Upper CI	98
Lower CI	42	Lower CI	98

Table S1. Sensitivity and Specificity Computations for Meta Regression

S7. Summary of Computations for Deeks' Funnel Plots

This section shows the computations obtained for each Deeks' funnel plot. It contains the regression statistics and the number of observations needed in the funnel plot.

Regression Statistics	
Multiple R	0.485613813
R Square	0.235820775
Adjusted R Square	-0.528358449
Standard Error	0.128505842
Observations	3

Table S2. Regression Statistics (Cancer type)

Regression Statistics	
Multiple R	1
R Square	1
Adjusted R Square	65535

Standard Error	0
Observations	2

Table S3. Regression Statistics (Vivo)

Regression Statistics	
Multiple R	0.521334868
R Square	0.271790045
Adjusted R Square	0.150421719
Standard Error	0.049353033
Observations	8

Table S4. Regression Statistics (AI Methods)

Regression Statistics	
Multiple R	1
R Square	1
Adjusted R Square	65535
Standard Error	0
Observations	2

Table S5. Regression Statistics (Published Years)

Regression Statistics	
Multiple R	0.249819145
R Square	0.062409605
	-
Adjusted R Square	0.093855461
Standard Error	0.105219719
Observations	8

Table S6. Regression Statistics (All Studies)

S8. Deeks' Funnel Plot Computation of p-value

This section shows the computations obtained for the p value needed in the Deeks' funnel plot. p value plays a significant role in determining the heterogeneity of the data involved. p values below 0.05 suggest heterogeneity, as well as, p values above 0.05 suggest no heterogeneity.

ANOVA

	df	*SS*	*MS*	*F*	*Significance F*
Regression	1	0.005096037	0.005096	0.308594	0.677192271
Residual	1	0.016513751	0.016514		
Total	2	0.021609788			

Table S7. P-value of Deeks' Funnel Plot (Cancer type)

ANOVA

	df	*SS*	*MS*	*F*	*Significance F*
Regression	1	4.30699E-06	4.31E-06	#NUM!	#NUM!
Residual	0	0	65535		
Total	1	4.30699E-06			

Table S8. P-value of Deeks' Funnel Plot (Vivo)

ANOVA

	df	*SS*	*MS*	*F*	*Significance F*
Regression	1	0.005454512	0.005455	2.239382	0.185172562
Residual	6	0.014614331	0.002436		
Total	7	0.020068843			

Table S9. P-value of Deeks' Funnel Plot (AI Method)

ANOVA

	df	*SS*	*MS*	*F*	*Significance F*
Regression	1	7.12536E-06	7.13E-06	#NUM!	#NUM!
Residual	0	0	65535		
Total	1	7.12536E-06			

Table S10. P-value of Deeks' Funnel Plot (Published years)

ANOVA

	df	*SS*	*MS*	*F*	*Significance F*
Regression	1	0.004421644	0.004422	0.399383	0.550713108
Residual	6	0.066427135	0.011071		
Total	7	0.070848779			

Table S11. P-value of Deeks' Funnel Plot (All Studies)

-
